# Dye decomposition and air de-pollution performance of TiO_2_/SiO_2_ and N-TiO_2_/SiO_2_ photocatalysts coated on Portland cement mortar substates

**DOI:** 10.1007/s11356-022-20228-8

**Published:** 2022-04-22

**Authors:** Souad Khannyra, Maria Luisa Almoraima Gil, Mohammed Addou, Maria Jesus Mosquera

**Affiliations:** 1 TEP-243 Nanomaterials Group, Department of Physical-Chemistry, Faculty of Sciences, University of Cadiz, 11510 Puerto Real, Spain; 2 Materials and Valorization of Natural Resource Laboratory, FST Tangier, Abdelmalek Essaadi University, Tétouan, Morocco

**Keywords:** N-TiO_2_/SiO_2_ composites, Clusters, Self-cleaning, NOx, Portland cement mortar, Air pollution

## Abstract

In this study, the newly synthesized TiO_2_ and N doped TiO_2_ clusters were added to silica sol to synthesize N-TiO_2_/SiO_2_ composites via the sol–gel method. Afterwards, the prepared sols were applied by brushing on portland cement. Doping with nitrogen significantly increased the absorption of TiO_2_ towards the visible region, thus, increasing the photocatalytic activity. SEM characterization of the treated samples showed that the clusters were distributed in form of aggregates on the samples’ surface. The self-cleaning and air de-polluting performances were assessed through methylene blue degradation and the oxidation of nitrogen oxide, resulting in methylene blue (MB) removal of 85% and 78% after 60 min of irradiation for SN10TiO_2_ and STiO_2_, respectively. Regarding air de-pollution performance, the newly synthesized photocatalysts showed the ability of NOx reduction. However, their efficiency was somewhat lower, in which 23.81% of NO has been oxidized by the sample SN10TiO_2_, while SP25 showed a total NO conversion of 38.98%. The powdered xerogels of the newly synthesized nanoparticles revealed high photocatalytic efficiency concerning NO oxidation, resulting in a higher performance compared to those obtained by the xerogel containing P25.

**Graphical abstract:**

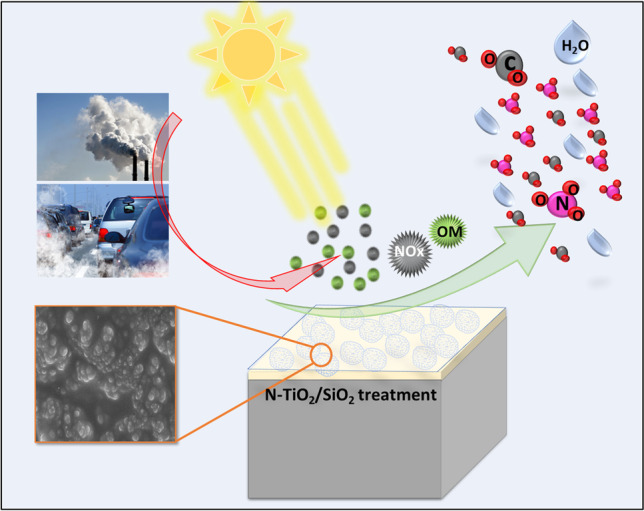

## Introduction

Air quality is a critical determinant of population health and well-being (Salgado et al. 2020). Particulate matter (PM) and nitrogen oxides (NOx) are among the most common urban pollutants, originating from various sources, mostly from industrial activities and the transportation sector. It has been proven that huge amounts of NOx and airborne are emitted annually in Europe (European Environment Agency 2014). These pollutants have a significant impact on human health and are the main cause of degradation of building materials. It is well known that NOx and soot released in the air promote cancer mortality, respiratory troubles and other diseases (WHO 2007; Kelly and Fussell 2012; Gasca-Sanchez et al. 2021). Moreover, these pollutants accumulate on building surfaces, causing staining and colour variation; additionally, these pollutants could dissolve in water (i.e. rain and surface condensation) and penetrate the pore of building surfaces, harming their aesthetic and structural aspect (Varotsos et al. 2009; Gibeaux et al. 2018), particularly, for portland cement mortar since it is one of the widely employed materials for building construction globally. Although considerable efforts have been made by industrial and transport technologies to minimize NOx emissions, its level is still too high, especially in large urban centres. One of the most advanced ways to remove specific pollutants from air is using photocatalysts as self-cleaning treatment for building material facades. A thin layer of semiconductors deposited on building surfaces can effectively diminish costs and increase the long-term aesthetic appearance of the subjected surfaces of building materials. Among the most used photocatalysts, TiO_2_ has been considered the suitable choice due to its high photocatalytic efficiency, chemical stability, inexpensiveness and non-toxicity (Noman et al. 2019; Korošec et al. 2020; Wongaree et al. 2022). Titanium dioxide is the most widely applied photocatalyst in self-cleaning construction materials and air remediation due to its high photocatalytic efficiency (Bergamonti et al. 2014; Singh et al. 2021; Khannyra et al. 2022).

Nevertheless, the photocatalytic efficacy of TiO_2_ is quite limited, mainly due to the lack of visible light absorption and the fast recombination of the photogenerated charges within TiO_2_ particles, and, consequently, its self-cleaning ability only occurs at 3–5% of the solar spectrum, i.e. the UV region of sunlight (Pelaez et al. 2012). Great efforts have been made to increase the absorption of TiO_2_ in the visible range and reduce the recombination of the photogenerated electron/hole pairs, like loading of noble metal (Au, Pd and Pt) (Fujimoto et al. 2017; Torras-Rosell et al. 2017; Yu et al. 2021). Although noble metals have been extensively used and recognized as good cocatalysts for improving the photocatalytic activity of titanium dioxide, these cocatalysts are scarce metals that are expensive to use, therefore, are not the appropriate choice for large-application, as in the case of building materials. Thus, doping titania with low-cost elements is one of the most effective methods to extend its response towards the visible region (Xu et al. 2008; Xu and Zhang 2010; Lv et al. 2013; Ananpattarachai et al. 2016; Bakar and Ribeiro 2016; Zhang et al. 2018). Among these elements, N was widely studied as a dopant for TiO_2_ due to its high abundance and low cost. Since Asahi and coworkers reported that N doping results in band gap red shift (Bracco et al. 2020), considerable efforts have been made to prepare N-doped TiO_2_ with the photocatalytic activity in the visible light region (Li et al. 2016; Biswas et al. 2018; Calisir et al. 2020; Xu et al. 2021). The use of these photocatalysts for outdoor applications cannot guarantee long-term performance due to their poor adherence to building materials substrates; for this reason, a matrix that ensures good adherence for long-term performance is needed. Silica has been considered an appropriate matrix for TiO_2_ immobilization that guarantees good adherence, therefore, preventing TiO_2_ release from building surfaces (Truppi et al. 2018), leading to the formation of durable materials (Pinho et al. 2013; Liu and Liu 2016; Khannyra et al. 2022).

Herein, new materials TiO_2_/SiO_2_ and N-TiO_2_/SiO_2_ have been synthesized via sol–gel method and applied by brush on the substrates to produce self-cleaning coatings for portland cement. To the best of our knowledge, there has been no report on applying N-TiO_2_/SiO_2_ as a self-cleaning coating for building materials substrates. A number of N-TiO_2_/SiO_2_ photocatalysts have been synthesized by varying the concentration of nitrogen doping in order to figure out its effect on the photocatalytic activity. The newly synthesized photocatalysts showed high self-cleaning behaviour and, the presence of nitrogen greatly enhanced their effectiveness. The self-cleaning performance of the coated samples has been evaluated employing two pollutants: first, the degradation of MB as a model dye; second, the oxidation of NO as a common pollutant released by industrial and transport activities in urbane areas. The TiO_2_ and N-TiO_2_ photocatalysts employed in this study are in the process of being published in the submitted patent number P202131136.

## Material and method

### Photocatalysts synthesis and application

The photocatalytic sols were manufactured as follow: first, N-TiO_2_ photocatalysts were synthesized via sol–gel method, in which titanium isopropoxide and urea precursors were used as the source of TiO_2_ and nitrogen doping. First, 10 ml of Ti-isopropoxide was added dropwise to 100 ml of 96% ethanol under vigorous stirring. The stirring was continued at 60 °C for 1 h; then, urea solution was added dropwise to the mixture followed by the addition of 10 ml of water, and stirring was continued for a further 3 h at 60 °C. Different urea solutions were prepared at concentrations of 0, 3.33, 6.66, 8 and 10 M. In order to study the effect of soaking in nitrogen, the synthesis was carried out without the employment of any reducing agent or stabilizing agent. Undoped TiO_2_ photocatalyst was synthesized following the same procedure without adding urea precursor. The obtained suspensions were washed several times with water and dried overnight at 90 °C. The powders were granulated and calcined at 500 °C at the ramp of 5 °C/min for 1 h. Second, the required amount of the previously synthesized TiO_2_ and N-TiO_2_ photocatalysts has been added to 30 ml of silica precursor, TES40 WN (Wacker, Munich, Germany), an ethoxysilane oligomer that has an average degree of polymerization 5. This precursor affords about 41% of silica after complete hydrolysis. The mixture was sonicated in an ultrasonic bath for 20 min to obtain better dispersion. After that, water and n-octylamine (catalyst, Sigma-Aldrich, St. Louis, MO, USA) have been added. The sols were mixed by using a Sonopuls HD3200 ultrasonic homogenizer from Bandelin (Berlin, Germany) under high-power agitation of 60 W for 10 min. The employed amount of water and n-octylamine respecting to silica oligomer was 0.83% v/v and 0.36% v/v, respectively. The photocatalyst proportion concerning silica oligomer was 4% w/v, and their formulation was as follow: matrix, STiO_2_, SN3.33TiO_2_, SN6.66TiO_2_, SN8TiO_2_ and SN10TiO_2_, for S0N0TiO_2_, TiO_2_, 3.33 N-TiO_2_, 6.66TiO_2_, 8 N-TiO_2_ and 10 N-TiO_2_, respectively. P25 photocatalyst was also employed in this study for further comparison.

The synthesized products were applied on portland cement mortar substrates by brushing to saturation three times; sols extra was removed by paper in order to obtain the thinnest possible layer.

The substrates employed in this study are portland cement mortar, consisting of CEMI/B-L 32.5R. The cement/sand and water/cement ration used in its formulation were 1:3 and 0.52, respectively. These samples have an open porosity of 14.6%; 80% of the total pore volume have a size ranging between 1 and 10 µm, calculated by mercury intrusion porosimetry (PIM) equipment.

### Sol–gel characterization

The viscosity of the prepared sols was evaluated immediately after the synthesis, using a concentric cylinder viscometer (model DV-II + with UL/Y adapter) from Brookfield (Middleborough, MA, USA). The experiments were carried out at an adjusted temperature of 30 °C, maintained by recirculated water from a thermostatic bath. The shear stress versus flow curve was generated.

The xerogels under study were obtained by depositing 10 ml of the prepared products in plastic Petri dishes that have a diameter of 85 mm and kept at room temperature (17 °C) till the gels were dried and after that were powdered and characterized as follow:

The UV–visible reflectance spectra were recorded using a Shimadzu UV-2600 spectrophotometer equipped with an ISR-2600 integrating sphere; BaSO4 powder was used as a white reference.

The photocatalysts structure and dispersion into silica matrix were also assessed; TEM/STEM in a Talos F200X, a focus ion beam (FIB) microscope with a maximum accelerating voltage of 200 kV, was employed for this purpose. This apparatus contains a HRSTEM scanning mode of 0.16 nm, with four detectors, high-angle annular dark-field (HAADF), ADF, DF and BF, that could be used simultaneously and 0.25 nm for the high-resolution transmission electron microscopy (HRSTEM) mode. The powdered xerogel was deposited on a lacey carbon coated copper grids.

For further insight, the textural properties of the synthesized nanoparticles were investigated by nitrogen physisorption experiments (Micromeritics ASAP2010, Norcross, GA, USA), working at 77 K and equipped with pressure transducer resolution of 10–4 mm Hg. Textural properties were calculated, considering BET and BJH standard models for the analysis. Before the assessment, the nanoparticles were degasified at 200 °C for 20 h.

### Self-cleaning and air de-pollution properties

A solid reflection spectrophotometer, Colorflex model from HunterLab, was employed to evaluate the colour changes induced by the treatment. The conditions used were illuminant D65 and observer 10°. CIELa*b* colour space and colour changes were assessed using the parameter total colour difference (ΔE*) (2000).

Surface topography of the treated samples and their untreated counterparts was visualized by scanning electron microscopy (SEM), using a Nova NanoSEM 450 model microscope from FEI Company. The secondary electron images were developed at an acceleration voltage of 5 kV and high vacuum conditions by means of a through the lens detector (TLD). Before that, the samples were metallized with a gold layer of 9 nm of thickness for preventing the sample charging.

Scotch® MagicTM tape (3 M) was used to carry out the peeling test in order to assess the adhesion of the coating on the substrates. The test was carried out by sticking a piece of adhesive tape on the surface of the samples and determining the increase of the weight of the tape after its detachment.

The self-cleaning efficiency of the treated samples was evaluated as follows: a solution of methylene blue of 1 mM was dissolved in ethanol; afterwards, 20 µl/cm^2^ of this solution was dropped on the surface of the treated samples and their untreated counterparts. Prior to the test, samples were kept in dark for 30 min to reach their equilibrium. Then, the samples were illuminated in a solar degradation chamber (Solarbox 3000eRH from CO.FO.ME.GRA), equipped with a 2500 W xenon arc lamp and an outdoor UV filter. Integrated detectors controlled and monitored the temperature, humidity and irradiance (in the range of 300–800 nm). The test of photocatalytic activity was performed under an irradion of 300 W/m^2^ of irradiance, temperature of 50 °C and an absolute humidity of 60 mg/m^3^. The progress of diffuse reflection spectra throughout the illumination was followed by using the previously described spectrophotometer. To determine the amount of MB removed, Kubelka–Munk function was employed to obtain the equivalent MB absorbance.

The effectiveness of these treatments was also evaluated through the oxidation of NOx for air de-pollution. The sample size of 10 × 5 × 2 cm^3^ was used for this study as stated by ISO 22197–1 (2016). Prior to testing, samples were irradiated with 1mW/cm^2^ of UV-A and *t* ≥ 5 h (overnight) to remove all existing organic materials on their surfaces; after that, the samples were washed with distilled water and dried under laboratory conditions (17 °C). The quantity of NO conversion, NOx removed, NO_2_ generated and the selectivity of NO_2_ generated were calculated employing the following expressions:

1 $${\mathrm\eta}_{\mathrm{NO}}=\frac{\mathrm f}{2.44}\int_{{\mathrm t}_{\mathrm{on}}}^{{\mathrm t}_{\mathrm{off}}}{\lbrack NO\rbrack}_{in}-{\lbrack NO\rbrack}_{\mathrm{out}}\mathrm{dt}$$

2 $${\mathrm\eta}_{{\mathrm{NO}}_2}=\frac{\mathrm f}{2.44}\int_{{\mathrm t}_{\mathrm{on}}}^{{\mathrm t}_{\mathrm{off}}}{{\lbrack\mathrm{NO}}_2\rbrack}_{\mathrm{out}}\mathrm{dt}$$

3 $${\mathrm\eta}_{{\mathrm{NO}}_{\mathrm x}}={\mathrm\eta}_{\mathrm{NO}}-{\mathrm\eta}_{{\mathrm{NO}}_2}$$

4 $$\%{\mathrm{NO}}_2\;\mathrm{selectivity}=100\frac{{\mathrm\eta}_{\mathrm{NO}2}}{{\mathrm\eta}_{\mathrm{NO}}}$$

where *ƞ*_xx_ are the μmols of gas removed or generated, *f* is the normalized air flow in l/min, and *t*_on_ and *t*_off_ are the times when the lamp switched on and off, respectively. [NO]_in_ represents the concentration of NO in the feed stream, and [NO]_out_ and [NO_2_] _out_ represent the concentration in ppm of NO or NO_2_ in the outlet stream, respectively.

## Results and discussion

### Sol–gel characterization

The rheological properties of the synthesized sols are an important parameter for the penetration and distribution of products in the substrate pore structure. The viscosity of the prepared sols was measured immediately after the products were synthesized. The obtained results of the studied samples are listed in Table 1. All the synthesized products revealed a Newtonian behaviour in the evaluated shear range. The values of the viscosity were determined as the slope of the shear stress versus shear rate curves. In all cases, the linear regression coefficients were above 0.99.

**Table 1:** Viscosity and gel time of the synthetized sols and textural properties of the studied xerogels

Sample	Viscosity	*R*^2^	*S*_total_ (m^2^/g)	*V*_pore_ (nm)
Matrix	4.87	0.99	496.03	2.47
SP25	6.38	0.99	480.03	2.83
STiO2	5.44	0.99	376.25	3.13
SN3.33TiO2	5.6	0.99	449.68	2.58
SN6.66TiO2	5.22	0.99	454.78	2.66
SN8TiO2	5.71	0.99	453.42	2.46
SN10TiO2	5.73	0.99	487.00	2.60

Silica-free nanoparticle sol showed a viscosity value very close to that of commercial silica sols (TES40, 4.82 mPas); by adding the TiO_2_ and N-TiO_2_ NPs to the sols, the viscosity value of the products slightly increased to become about 5.5 mPas. The product containing P25 NPs showed a viscosity value somewhat higher compared to the newly synthesized NPs. There is a clear relationship between the viscosity and volume concentration, in which the viscosity increases with the concentration. Previous studies have observed similar behaviour, which links the viscosity and volume concentration (Pinho et al. 2015). From the obtained values, it is clear that nitrogen doping did not affect the rheological properties. The viscosity value was practically similar for all the products containing TiO_2_ and N-TiO_2_ NPs. Thus, these findings allow us to assume that all the synthesized products could penetrate into mortar substrates similarly to commercial silica, providing good spreading of the sols throughout the porous structure of the substrates.

Gel time is another important parameter for building materials that plays an important role, especially for in situ applications. Immediate gelation strongly affects the product penetration in the pores of the substrates, inhibiting their penetration, especially when these products are applied in situ. Part of this products was stored in closed bottles, and the second part was deposited in plastic Petri dishes in order to boost the spontaneous sol–gel transitions. This latest demonstrated that the sol–gel transitions took place in a few hours, resulting in a homogeneous crack-free gigantic molecule. No remarkable difference in sol–gel transition has been observed, in which all the products showed overnight sol–gel transitions, indicating that the presence of the photocatalytic nanoparticles did not affect the gelation time, whereas the products stored in closed bottles were remained as sol after 1 year of storage.

**Fig. 1:**
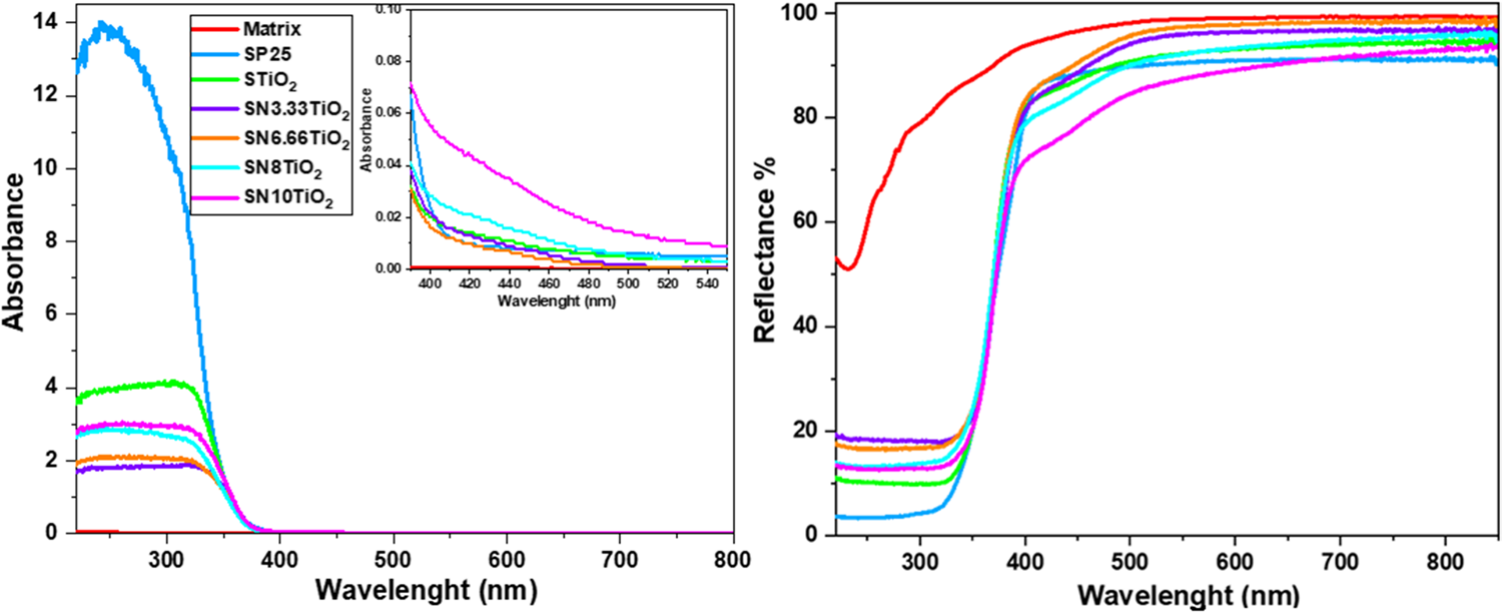
Diffuse reflectance UV–visible absorbance and reflectance spectra of the xerogels under study

To investigate the optical absorption of the synthesized products, UV–visible diffuse reflectance has been carried out. Figure 1 shows Kubelka–Munk transformation of diffuse reflectance and reflectance spectra of the xerogels under study, recorded in the wavelength range of 220–800 nm. It is seen that the photocatalyst-loaded nitrogen showed a considerable increase in the visible range absorption in comparison to the products containing nitrogen-free TiO_2_ and P25. Moreover, the intensity of visible absorption was raised with nitrogen loading. These findings indicate that the sensitivity of the photocatalysts towards the visible light illumination has been increased and at the same time evidenced the presence of nitrogen within TiO_2_ network, enhancing the photocatalytic activity as will be discussed in the following sections.

**Fig. 2:**
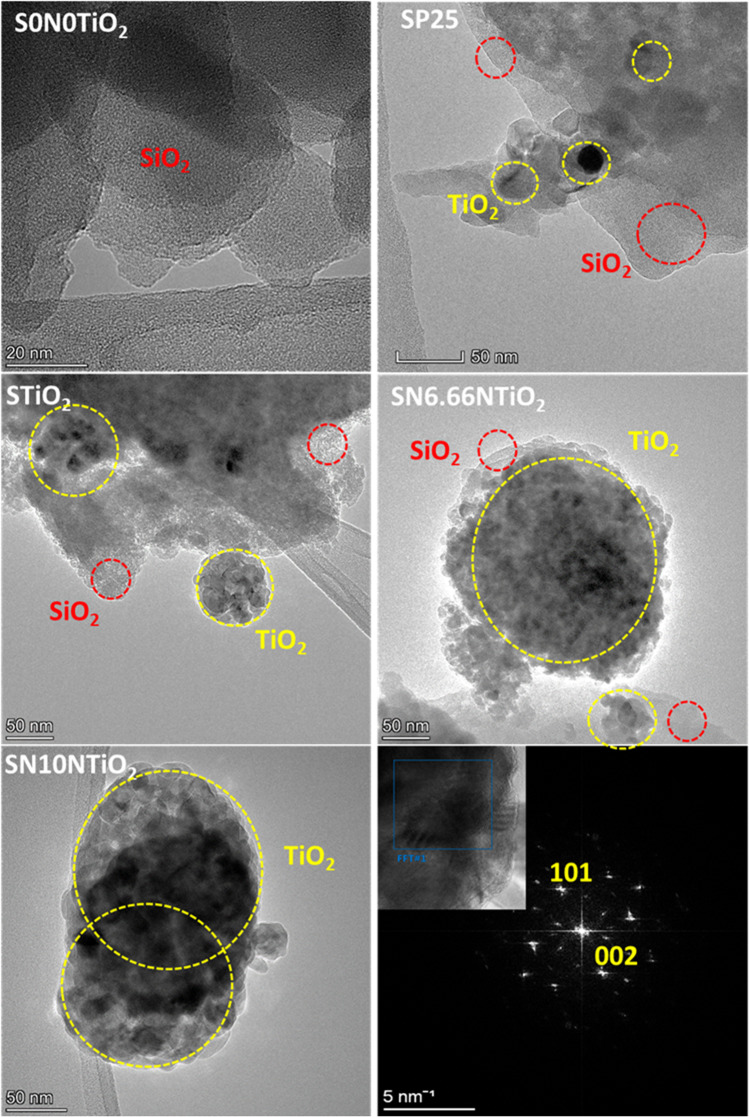
TEM images of the studied photocatalysts and high-resolution (HRTEM) image of 10 N-TiO_2_ and its fast Fourier transformation (FFT) diagram

The dried xerogels were powdered and characterized by TEM to figure out further details on their morphology and photocatalyst distribution into the silica matrix. Figure 2 illustrates the representative TEM micrographs of SP25, STiO_2_, SN6.66TiO_2_ and SN10TiO_2_ photocatalysts. These samples were selected as a model to explore the size and shape of the synthesized nanoparticles. At first sight, all the photocatalysts consisted of amorphous silica particles, TiO_2_ particles and TiO_2_ aggregates of P25 and the aggregated TiO_2_ and N-TiO_2_ clusters. In addition, it is evident that the P25 NPs were incorporated into silica in the form of separated nanoparticles and small aggregates, while it seems clear that the newly synthesized photocatalysts were integrated forming individuals and aggregated clusters, with sizes ranging from 50 to 200 nm, indicating that the synthesis method does not alter the shape of the cluster.

The fast Fourier transformation (FFT) diagram corresponding to the photocatalyst 10N-TiO_2_/SiO_2_ is given in the last images, where the inset images shows the crystalline plans. The presence of the lattice planes (101) and (002) confirms the anatase phase crystalline structure of the photocatalysts.

**Fig. 3:**
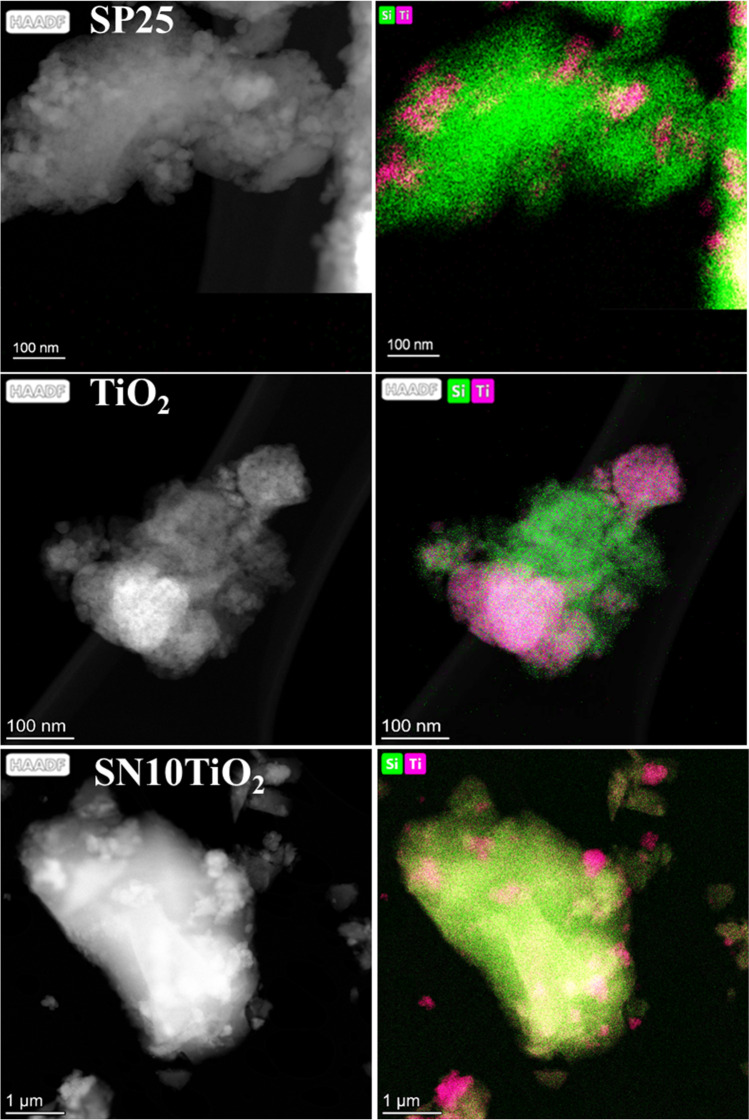
STEM-HAADF images and their corresponding XEDS maps of the studied photocatalysts

For better insight into the distribution of the photocatalysts in the silica matrix, STEM-HAADF mode was employed for this purpose. Figure 3 illustrates the HAADF images of the characterized samples. It is found that all the photocatalysts revealed aggregates homogeneously distributed throughout the silica matrix. However, the size of aggregates was different, in which P25 NPs exhibited gathers of size less than 100 nm, while the TiO_2_ clusters showed various size distributions, indicating that the clusters were incorporated in the form of individuals and aggregates as previously observed by TEM images.

**Fig. 4:**
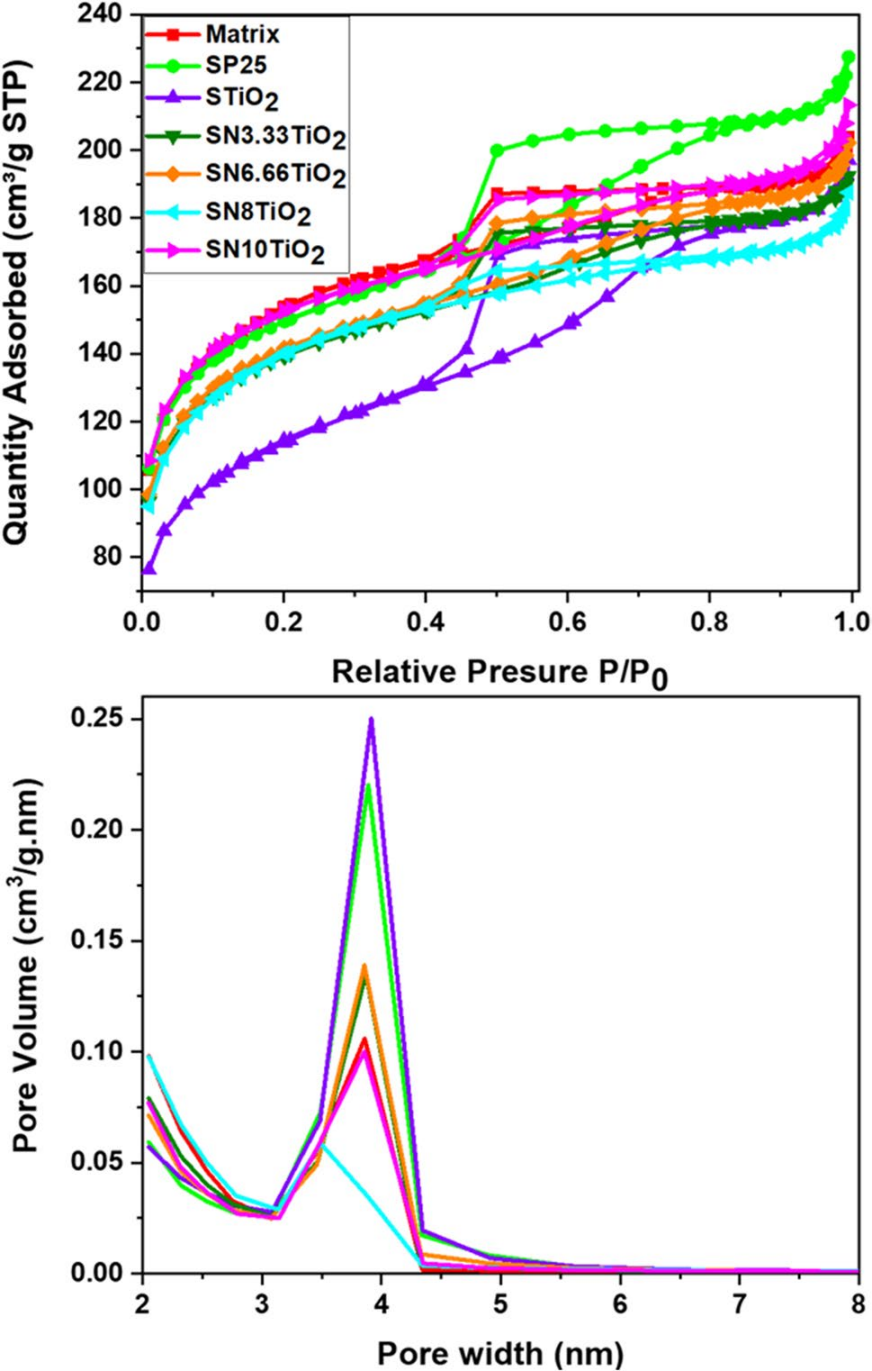
Adsorption–desorption isotherms and pore size distributions of the powdered xerogels

To better understand the relationship between the textural properties and the photoactivity of the manufactured photocatalysts, N_2_ physisorption was carried out. The obtained isotherms and the pore size distribution are given in Fig. 4. According to the IUPAC classification, all photocatalysts exhibited type IV isotherms (Fig. 4, top), which are characteristic of mesoporous materials. Moreover, the photocatalysts showed an H_2_-type hysteresis loop, characterized by a triangular shape and a steep absorption branch, indicating that the pore structure consists of interconnected networks of different sizes and shapes.

Figure 4 (bottom) shows the pore size distribution of the manufactured photocatalysts obtained from the isotherm desorption branches. All the samples showed practically similar pore size distribution, micropores of size lower than 2 nm, and mesopores vary between 3 and 5 nm, with a maximum around 4 nm. However, some difference between them has been observed, in which the photocatalysts SP25 and STiO_2_ revealed the highest pore volume distribution, while the photocatalysts containing nitrogen showed somewhat lower pore volume. In addition, the photocatalyst SN8TiO_2_ exhibited the lowest pore volume distribution with a maximum pore size distribution of about 3.5 nm.

The BET surface area values were practically similar for almost all the photocatalysts, except the photocatalysts STiO_2_ that showed a somewhat lower surface area of a value of 376.25 m^2^/g. In comparison, the rest of the samples revealed a surface area of 450 to 480 m^2^/g.

### Application on mortar materials and the evaluation of the photocatalytic activity

The synthesized products were applied by brush on Portland cement mortar substrates in order to investigate their adherence, self-cleaning and air de-pollution properties. A photocatalyst-free coating sample (S0N0T) was also synthesized and applied for further comparison.

The uptakes of the applied products on mortar substrates was recorded, and their values are listed in Table 2. It should be noted that there is a clear relationship between viscosity and the uptake. Since the viscosity value of the synthesized products was very similar, the uptake values were also quite similar, with no remarkable difference.

**Table 2:** The uptake values, colour change induced by the coatings and the weight of the material removed by peeling test

Sample	Uptake (g/m^2^)	ΔE*	Removed material × 10^−5^ (g/cm^2^)
Untreated	-	-	6.57
Matrix	399.25 ± 43.66	4.99 ± 0.77	< 0.01
SP25	429.33 ± 25.21	7.55 ± 1.62	< 0.01
STiO_2_	452.28 ± 83.30	7.91 ± 1.23	< 0.01
SN3.33TiO_2_	437.61 ± 37.41	7.50 ± 0.40	< 0.01
SN6.66TiO_2_	385.41 ± 9.52	6.89 ± 0.69	< 0.01
SN8TiO_2_	435.78 ± 49.77	7.22 ± 0.93	< 0.01
SN10TiO_2_	428.40 ± 33.27	6.15 ± 1.12	< 0.01

The treatment’s colour change is a key factor, particularly for historical heritage. After measuring the total colour change ΔE*, it has been observed that the applied treatments induced significant colour changes on the substrates, exhibiting a total colour change ΔE* > 5 for all the photocatalytic coatings. This change of colour is due to the nature of the original colour of the substrates (grey) and that of the products (beige).

**Fig. 5:**
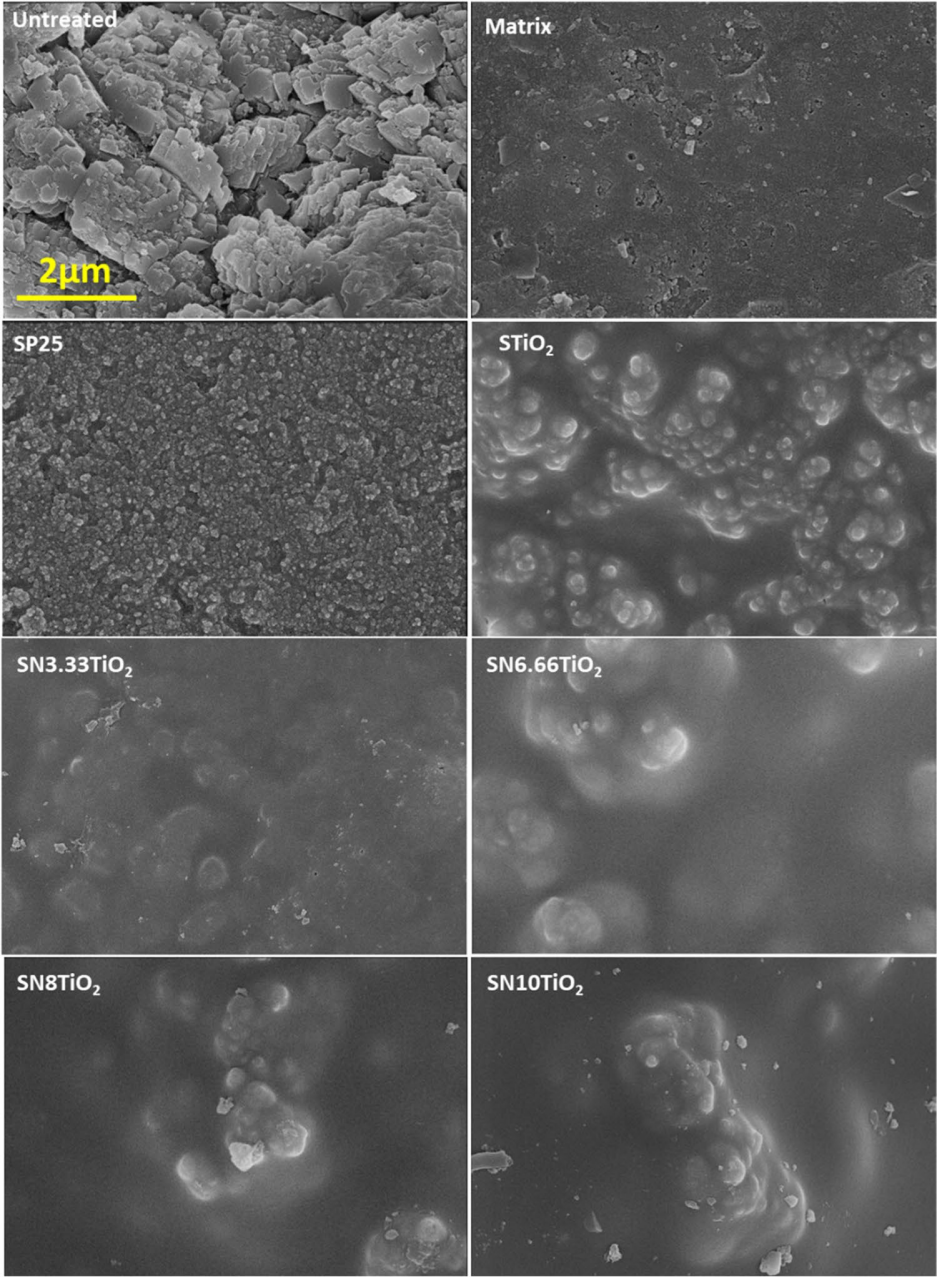
SEM images of the treated samples and their untreated counterparts. The scale bar for untreated sample universally applicable for all sub-figures

The treated samples and their untreated counterparts were characterized by SEM in order to figure out the effect of the coatings on the topography of the surface. The obtained micrographs are given in Fig. 5. At first glance, it has been observed that the presence of the treatments significantly reduced the roughness of mortar substrates, forming smooth surfaces compared to the untreated sample that revealed a rougher surface resulting from different sizes of mortar material grains. This smoothness is a consequence of the presence of silica as a main component of the coatings, which led to the formation of coating’s surfaces with less roughness and helps develop well-adhered and durable treatments. It is also important to note that all treatments have exhibited homogeneous crack-free coatings. This behaviour has been previously observed in our earlier work (Khannyra et al. 2021), which is an outcome of n-octylamine surfactant that plays an essential role in coating adhesion and the formation of crack-free coatings (Illescas and Mosquera 2011).

Regarding the topography of the samples treated with the products containing the synthesized clusters, it has been seen that all of them have shown rougher surfaces compared to those treated with the photocatalysts-free silica and SP25, where distinctly sized aggregates with different levels were observed, forming uneven coatings. These aggregates are evidentially related to the clusters and their aggregations. Moreover, it is also observed that these aggregates are covered by silica gel compared with the samples treated by the coating containing P25, which exhibited dispersed nanoparticles throughout the entire surface of the substrate. The fact that the clusters were entirely covered by silica gel might be a drawback for the photocatalytic activity, particularly for nitrogen oxide reduction.

A peeling test was carried out to assess the adhesion of the treatments to the substrates. Excellent adhesion enables the development of durable coatings, especially for outdoor applications. The weight loss of the untreated samples and their treated counterparts are listed in Table 2. The obtained data clearly indicated that the treatments significantly reduced the amount of removed material. Their values were under the sensibility of the method (their value being practically zero). The mass removed from the untreated samples was significantly greater than that of the treated samples (6.57 × 10^−5^). These findings indicate that these treatments can be employed as durable coatings for outdoor applications. This result accords with our earlier work, where the study was evaluated on concrete substrates (Khannyra et al. 2021).

**Fig. 6:**
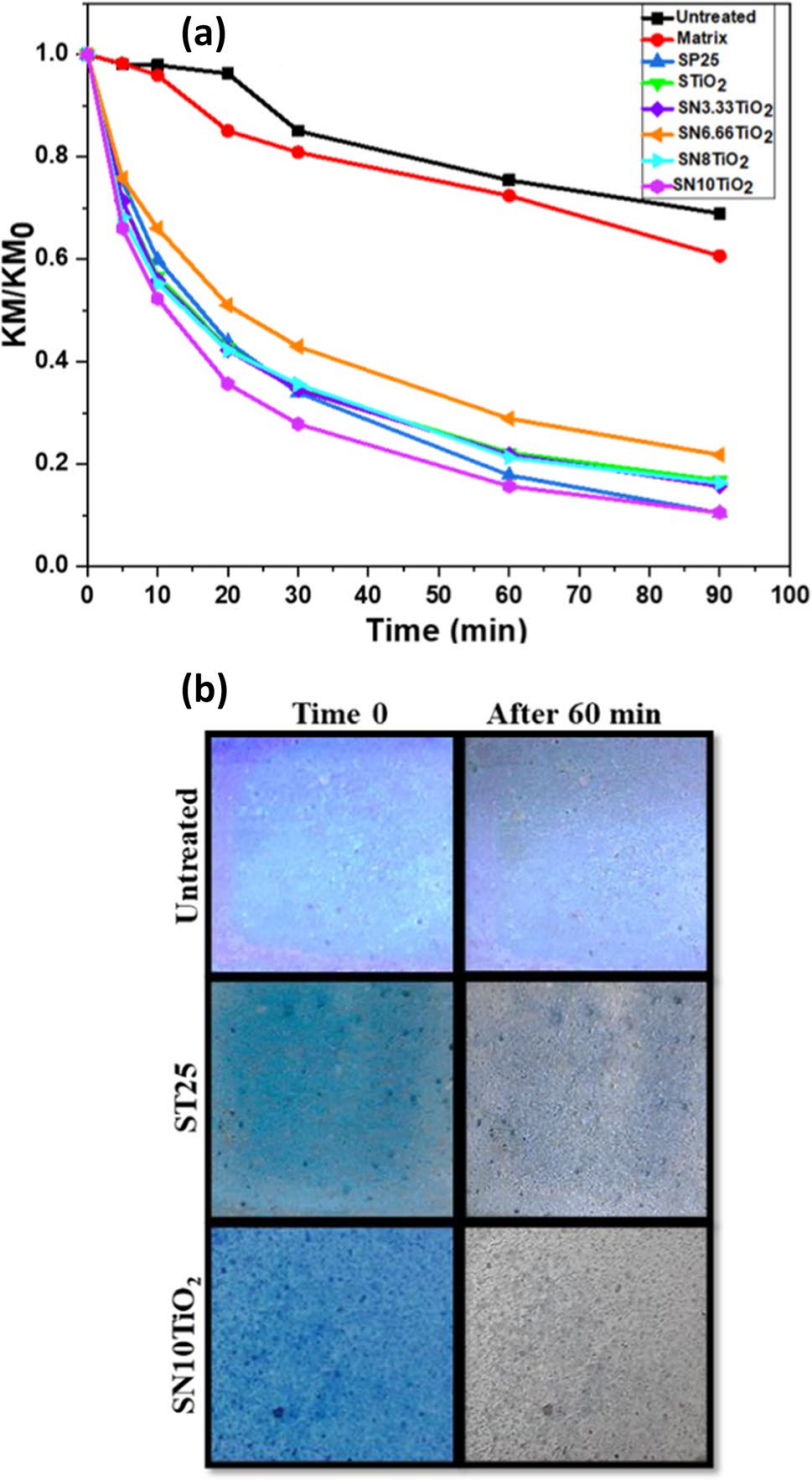
(**a**) Evolution of MB photodegradation and (**b**) photographs showing the evolution of the untreated, SP25 and SN10TiO_2_ samples, stained with MB during the time 0 and after 60 min of irradiation

Photocatalytic efficiency of the deposited photocatalysts on mortar substrates was evaluated through methylene blue degradation. The results of the evolution of stained surfaces with MB under UV–vis irradiation are given in Fig. 6a. As expected, a small amount of MB degradation has been observed for the untreated samples and those treated with TiO_2_-free silica (S0N0T), in which the percentage removed was around 25% and 27% after the first 60 min of irradiation for the untreated samples and S0N0T, respectively. This degradation is often due to the sensitivity of MB to UV–vis light.

Regarding the samples treated with the products containing TiO_2_ and N doped TiO_2_ photocatalysts, these coatings showed high efficiency, and their photocatalytic trend was as follow: SN10TiO_2_ > SP25 > SN8TiO_2_ > SN3.33TiO_2_ > STiO_2_ > SN6.66TiO_2_, reaching a degradation rate of MB of 85%, 83%, 79.5%, 79%, 78% and 72%, respectively, after 60 min of irradiation. These results proved the effectiveness of the newly synthesized photocatalysts as coatings on building materials for removing organic pollutants, providing high photocatalytic efficiency. Moreover, the sample with higher nitrogen loading exhibited the greatest kinetic of degradation in this study, leading to somewhat higher performance than the commercial P25. By comparing the efficacy of these coatings, the kinetics of degradation was increased with the amount of nitrogen added. In contrast, the coatings SN6.66TiO_2_ revealed a marginally lower kinetic of degradation than other coatings.

Despite the lower surface area of the photocatalyst STiO_2_, this sample exhibited high pore volume and similar pore size to the other photocatalysts, which could be the main reason behind its high photocatalytic performance. The photocatalyst SN8TiO_2_ revealed a small pore volume and pore size compared to the rest of the photocatalysts. On the other hand, this sample has a similar surface area to other photocatalysts; the photocatalytic activity increases with the rise of surface area, which could explain its high photocatalytic efficiency. In general, the main reason behind the increase of the photocatalytic activity is that the introduction of nitrogen extended the absorption towards the visible region; therefore, high photocatalytic efficiency has been produced.

Figure 6b shows the colour change of the untreated sample and those treated with the photocatalysts SP25 and SN10TiO_2_, before and after 60 min of UV–vis irradiation. These images clearly indicated that the untreated sample remained stained after 60 min of irradiation, and no remarkable difference was observed between the irradiated and non-irradiated samples. On the other hand, the stain on the sample treated by the photocatalyst SP25 was almost totally removed after 60 min of irradiation. A minimal amount of the stain remains on the surface. By contrast, the stain on the sample treated with the photocatalyst SN10TiO_2_ was totally bleached after 60 min of UV–vis light irradiation, confirming the high photocatalytic efficiency of this sample.

**Fig. 7:**
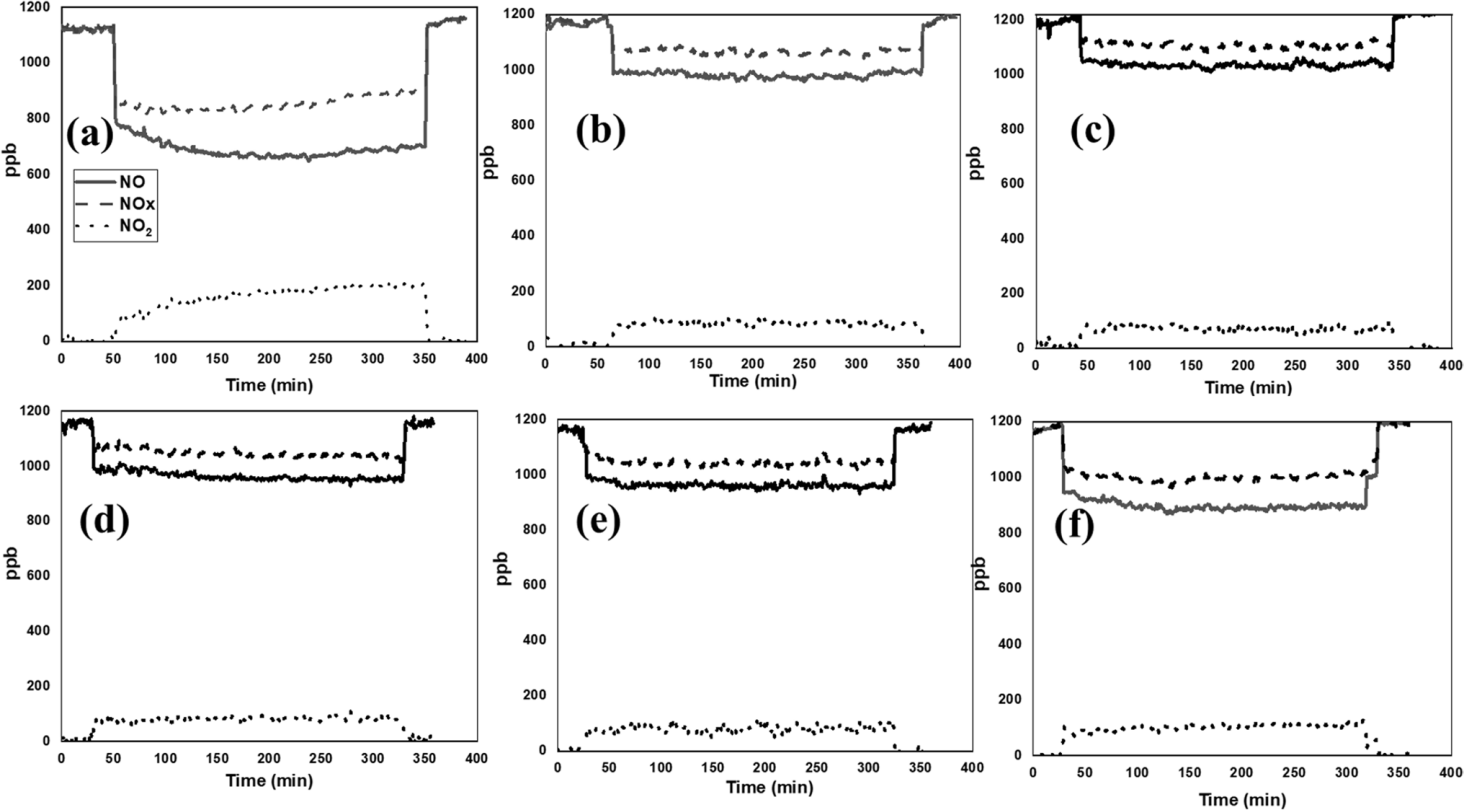
Evolution of NO, NO_2_ and NOx concentration profiles on the treated substrates during the 5 h of NO photodegradation tests under UV–visible irradiation: SP25 (**a**), STiO_2_ (**b**), SN3.33TiO_2_ (**c**), SN6.66TiO_2_ (**d**), SN8TiO_2_ (**e**) and SN10TiO_2_ (**f**)

Regarding the ability of the prepared coatings to reduce nitrogen oxides from the air, the treated samples and their untreated counterparts were exposed to UV light overnight to remove any existing organic compounds on their surfaces, then washed with distilled water and dried under laboratory conditions. The test was done following NOx ISO standard, wherein the samples were placed in a sealed reactor with no detectable leaks. The evolution profiles of NO, NO_2_ and NOx are presented in Fig. 7. Obviously, in the absence of irradiation, no photocatalytic activity can occur. The NO concentration remained constant, explaining that the photocatalytic sites are not activated. Once the UV–vis light turned on, the NO concentration fell sharply, reaching its maximum and remaining stable throughout the 5 h of the test, indicating the complete activation of the photocatalytic sites and their stability. At the same time, the concentration of NO_2_ was increased, indicating that the reaction between NO and O_2_ had started. Despite the increase in the concentration of NO_2_ generated, the amount of the total nitrogen oxides (NOx) removed remained stable. As soon as the light switched off, the NO, NO_2_ and NOx return to their initial values. The photocatalytic activity was also evaluated on the untreated sample for possible photolysis; the results revealed that no photooxidation has occurred. Similar results have been obtained by the sample treated with the photocatalyst-free coating (S0N0TiO_2_), pointing out that NOx could not be reduced without photocatalyst and at the same time confirming the ability of the synthesized photocatalysts in reducing air pollution (Silvestri et al. 2016; Chen et al. 2020).

By comparing the performance of the coatings containing the newly synthesized photocatalysts with that containing P25 NPs, it is clearly seen that the newly synthesized photocatalysts have the ability to oxidize NOx, although their efficiency was slightly lower than that of P25 NPs that exhibited a total NO conversion of ~ 39%, while the photocatalyst SN10TiO_2_ displayed a total NO conversion of ~ 24%. However, the selectivity of NOx removed by these photocatalysts was quite similar to that of SP25. The low performance of the newly synthesized photocatalysts might be due to the clusters being aggregated and were not homogeneously distributed throughout the substrate’s surfaces. Moreover, these photocatalysts were covered by silica gel compared to those of P25 that exhibited a particulate surface and nanoparticles homogeneously dispersed on the entire substrate (see SEM images in Fig. 5). It has been demonstrated that silica gel could absorb part of UV light (Yusof and Johan 2014; Ghorbanpour et al. 2017), preventing the contact light photocatalysts, therefore, decreasing the efficiency of photocatalytic oxidation processes of NO. In addition, silica gel can also impede the adsorption of nitrogen oxide on the photocatalytic surfaces, which could be one of the reasons behind the lower photocatalytic efficiency.

Concerning the influence of nitrogen, no significant difference has been observed between the undoped photocatalyst (STiO_2_) and those doped with nitrogen, for loading up to 8 M (SN8TiO_2_ photocatalyst), in which the total of NO removed was between 14 and 16%, except for the photocatalyst with higher nitrogen loading SN10TiO_2_ that revealed NO conversion somewhat higher (the amount of NO removed was up to 24%) compared to the previous photocatalysts (Table 3).

**Table 3:** Results of the test of NO photooxidation on the terated substrates

Sample	Gas supply vol. fraction (ppm)	% NO removed	% NO_2_ generated	% NOx removed	NOx selectivity	NO_2_ selectivity
Untreated	1165	-	-	-	-	-
Matrix	1170	-	-	-	-	-
SP25	1136	38.98	14.28	24.70	63.36	36.63
STiO2	1176	15.23	7.12	8.11	53.23	46.76
SN3.33TiO2	1212	13.98	5.81	8.16	58.40	41.60
SN6.66TiO2	1152	15.95	6.82	9.14	57.27	42.72
SN8TiO2	1162	13.98	5.81	8.16	59.05	40.95
SN10TiO2	1188	23.81	8.64	15.16	63.70	36.30

**Fig. 8:**
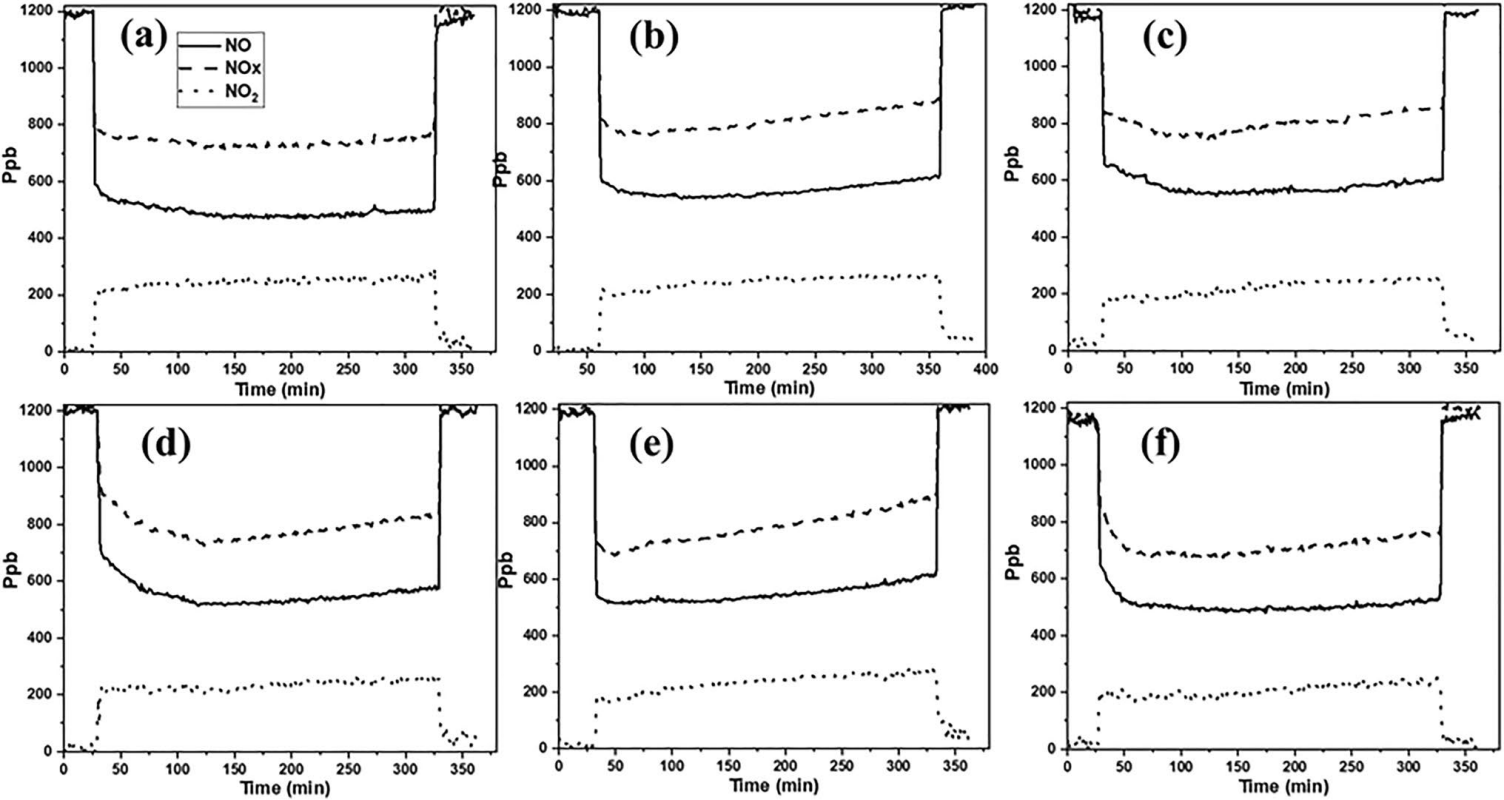
Evolution of NO, NO2 and NOx concentration profiles on the palettes of the powdered xerogels during the 5 h of NO photodegradation tests under UV–visible irradiation: SP25 (**a**), STiO_2_ (**b**), SN3.33TiO_2_ (**c**), SN6.66TiO_2_ (**d**), SN8TiO_2_ (**e**) and SN10TiO_2_ (**f**)

To better understand the photocatalytic behaviour of these photocatalysts and confirm the previous hypothesis, 1.5 g of the powdered xerogels was deposited in a holder of the same size as the substrates and pressed to form a homogeneous palette. After that, the holder was placed in the reactor, and the test of NOx reduction was carried out at the same conditions as previously done for the treated substrates. Figure 8 indicates the evolution of NO, NO_2_ and NOx profiles. As we expected, the photocatalysts revealed high photocatalytic performance, similar to that of the photocatalyst containing P25 NPs. By comparing the amount of NO removed, it is clearly seen that the photocatalyst SP25 showed somewhat higher NO removal 57.69% compared to the newly synthesized photocatalysts (see Table 4). However, this sample generates more NO_2_, which is also a harmful gas, indicating that the reaction of NO_2_ to HNO_3_ is slowed down; therefore, more NO_2_ is accumulated on the surface of the photocatalyst.

**Table 4:** Results of the test of NO photooxidation on the powdered xerogels palettes

Sample	Gas supply vol. fraction (ppm)	% NO removed	% NO_2_ generated	% NOx removed	NOx selectivity	NO_2_ selectivity
Matrix	1180	-	-	-	-	-
SP25	1177	57.69	18.70	38.99	67.58	32.41
STiO_2_	1201	52.69	17.82	34.88	66.19	33.80
SN3.33TiO_2_	1180	50.82	15.39	35.42	69.70	30.29
SN6.66TiO_2_	1198	53.35	16.90	36.45	68.32	31.67
SN8TiO_2_	1196	53.55	16.49	37.05	69.21	30.78
SN10TiO_2_	1160	55.73	15.93	39.80	71.41	28.58

Regarding the effect of nitrogen doping, it has been observed that the efficiency of the photocatalysts was increased with nitrogen doping. The NO conversion rates were 52.63%, 50.82%, 53.35%, 53.55% and 55.73% for TiO_2_, SN3.33TiO_2_, SN6.66TiO_2_, SN8TiO_2_ and SN10TiO_2_, respectively. Moreover, the presence of nitrogen raised the selectivity of NOx removal, wherein the selectivity has increased from 66.19 to 69.27% for nitrogen doping concentration up to 8 M. Concurrently, these samples witnessed a decline in the selectivity of the generated NO_2_, indicating that most of the generated NO_2_ has oxidized to HNO_3_. The greatest photocatalytic efficiency has been obtained by the sample SN10TiO_2_, which exhibited high NOx removal and lower selectivity of NO_2_ generated (see Table 4). These results are a consequence of nitrogen doping that revealed a great increase in the visible range, thereby preventing the recombination of the photogenerated electron/hole resulting in a significant enhancement of the photocatalytic activity. These findings confirm the previous hypothesis and, at the same time, underline the photocatalytic efficiency of these photocatalysts towards NOx reduction.

## Conclusion

In this investigation, the aim was to assess the self-cleaning and air de-polluting efficiency of the newly synthesized TiO_2_ and N-TiO_2_ clusters as coatings on Portland cement mortar substrates, wherein these clusters were integrated into silica matrix in order to ensure the good adherence and durability of the coatings onto the substrates. The characterization of the powdered xerogels showed that the doping with nitrogen significantly shifted the absorption towards the visible region. TEM characterization revealed that the clusters are distributed into silica matrix in the form of individual and aggregates; the latest was also observed on the surface of the substrates using SEM characterization. The adhesion test demonstrated that all the treatments were tightly adhered to Portland cement substrates, resulting in crack-free coatings.

The effectiveness of these coatings was evaluated through MB degradation and the oxidation of NO for air de-pollution. The results revealed that the treatments showed high photocatalytic activity, and, their performance was raised with nitrogen doping, in which 85% of the dye was destroyed after 60 min of irradiation by the sample containing the greater amount of nitrogen doping (SN10TiO_2_). However, these coatings showed a slightly lower efficiency concerning the oxidation of NO compared to the coatings containing P25 NPs, which must be a consequence of two factors: (i) the presence of aggregates on the surface of the treatment and (ii) the silica gel being totally covering the clusters. The assessment of the powdered xerogels demonstrated that the newly synthesized photocatalysts are highly photoactive and that their effectiveness was similar to that of the xerogel containing P25. Moreover, the xerogel with high nitrogen loading showed somewhat higher efficiency than that containing P25 photocatalyst.

## Data Availability

Not applicable.
