# Correction to: Dye decomposition and air de‑pollution performance of TiO_2_/SiO_2_ and N‑TiO_2_/SiO_2_ photocatalysts coated on Portland cement mortar substates

**DOI:** 10.1007/s11356-022-21198-7

**Published:** 2022-06-04

**Authors:** Souad Khannyra, Maria Luisa Almoraima Gil, Mohammed Addou, Maria Jesus Mosquera

**Affiliations:** 1 TEP‑243 Nanomaterials Group, Department, of Physical‑Chemistry, Faculty of Sciences, University of Cadiz, 11510 Puerto Real, Spain; 2 Materials and Valorization of Natural Resource Laboratory, FST Tangier, Abdelmalek Essaadi University, Tétouan, Morocco

**Correction to: Environmental Science and Pollution Research**

10.1007/s11356-022-20228-8

The image of Figure 7 is the same with Figure 8. It is modified in the original published proof.

The Original article has been corrected.

